# Inflammation-related cytokines and their roles in gastroenteropancreatic neuroendocrine neoplasms

**DOI:** 10.17305/bjbms.2020.4471

**Published:** 2020-11

**Authors:** Davorka Herman Mahečić, Maja Cigrovski Berković, Vanja Zjačić-Rotkvić, Tamara Čačev, Sanja Kapitanović, Monika Ulamec

**Affiliations:** 1 Department of Endocrinology, Diabetes and Metabolism, University Hospital Centre Sestre milosrdnice, Zagreb, Croatia; 2 Faculty of Kinesiology, University of Zagreb, Zagreb, Croatia; 3 Department of Molecular Medicine, Rudjer Boskovic Institute, Zagreb, Croatia; 4 Department of Pathology and Cytology Ljudevit Jurak, University Hospital Centre Sestre milosrdnice, Zagreb, Croatia; 5 Pathology Department, School of Medicine, University of Zagreb, Zagreb, Croatia

**Keywords:** Inflammatory microenvironment, TNF-α, IL-6, IL-1β, IL-2, GEP-NEN

## Abstract

Proinflammatory counterworks are important at different stages of tumor development, particularly during invasion and metastasis. Immune cells and their signal molecules can influence all stages of tumor progression, as well as therapeutic intervention. Proinflammatory cytokines are known triggers of growth in gastroenteropancreatic neuroendocrine neoplasms (GEP-NENs). In this study, we explored the immunohistochemical expression of tumor necrosis factor alpha (TNF-α), interleukin 1 beta (IL-1β), IL-2, and IL-6 in tissues from 43 GEP-NEN patients with tumors of gastric, duodenal, ileal, appendiceal, and colonic origin. The immunohistochemical expression of TNF-α was increased in tumor groups with high proliferation rates (Ki-67; *p* = 0.034), as well as in those with higher tumor grades (*p* = 0.05). Moreover, the immunohistochemical expression of TNF-α positively correlated with death outcomes (*p* = 0.016). Expression of IL-6, IL-1β, and IL-2 displayed similar immunohistochemical expression patterns regardless of Ki-67, although the expression between the ILs differed. Most GEP-NENs had high levels of IL-6 and lower levels of IL-1β and IL-2. Although further comprehensive studies are required for a complete understanding of activated mechanisms in proinflammatory protumoral microenvironment of GEP-NENs, TNF-α is a potential marker in the prognosis of those tumors.

## INTRODUCTION

Inflammation is one of the mechanisms involved in creating protumoral microenvironments in many different organs, in particular, in the gastrointestinal tract. In normal tissue remodeling, chronic inflammation is terminated when repair is finished. In the tissues with carcinogenic mutations, compartments surrounding the epithelial component of tumor, cancer-associated fibroblasts, immune and inflammatory cells, as well the vascular network and lymphatic spaces interact to create protumoral environments. Cytokines play an important role in the communication between epithelial cells and other compartments; disruption of this communication can lead to intratumoral tissues and peritumor parenchyma changes. The exact mechanisms creating such proinflammatory environments, as well as how they influence the growth and promotion of tumors, are still not entirely clear [1-3].

Several cytokines such as tumor necrosis factor alpha (TNF-α), interleukin (IL)-1α, IL-1β, IL-6, IL-10, IL-12, IL-17, and IL-23 have been identified as important messengers within the aforementioned environment. They have an important role in regulating inflammatory cells, especially macrophages, to create protumoral microenvironments during chronic inflammation and provide the tumor with the ability to evade host responses. TNF-α, IL-6, and IL-17 have known roles in tumor growth and promotion; IL-6 and IL-10 are produced by tumor-associated macrophages and create conditions of immune suppression and angiogenesis. STAT3 pathways are activated by some of these cytokines, and their activation of malignant cells stimulates their proliferation and survival; indeed, upregulating cytokines IL-1β and IL-6 promotes angiogenesis and tumor invasion. The NF-κB pathways are important during cytokine activation as they regulate tumor cell survival during early tumor promotion and induce the transcription of genes for proinflammatory cytokines, such as TNF-α and IL-6. IL-1β damages inflammatory tissues and stimulates tumor invasion by creating a suitable microenvironment for angiogenesis. IL-2 is well known agent for derivating immune response in antitumor manner. These pathways are permanently activated, with the help of cytokines, when conditions for tumor growth and progression are provided [1,2]. Conversely, IL-2 is well-known to have antitumor activity via modulation of immune responses. In the colon, loss of p53 during tumor progression is related to the formation of an NF-κB-dependent inflammatory microenvironment. Together, these pathways participate in the mechanisms behind inflammatory bowel disease increasing the risks of colorectal cancer, as well as the risks of gastrointestinal neuroendocrine tumors [4,5].

The cells of the diffuse endocrine system which are found in mucosa of the gastrointestinal tract are the cells of origin for gastroenteropancreatic neuroendocrine neoplasms (GEP-NENs), and are hyperstimulated by chronic inflammation. Proinflammatory cytokines IL-1 and IL-2 have been shown to mediate neuroendocrine differentiation in tumor cells and gastrointestinal hormone synthesis and secretion, respectively [6]. GEP-NENs are a non-homogeneous group of tumors, displaying a wide spectrum of aggressiveness. GEP-NENs have an estimated incidence of 3.6/100,000 and prevalence of 35/100,000. Although most have an indolent course, up to 40% of tumors have already metastasized by the time of diagnosis, most commonly in the liver and adjacent lymph nodes. However, even in the presence of liver metastases, some patients may survive for many years, suggesting that morphology and currently used grading/staging systems alone cannot predict tumor behavior.

Biomarkers that can identify tumors earlier, as well as providing an individualized selection of therapeutic treatments are currently under investigation globally [6-9]. The Ki-67 index is currently used as the most reliable proliferative marker of GEP-NENs as it independently correlates with survival; therefore, it is part of the grading system of tumors. Problems in assessing the Ki-67 index include intertumoral and intratumoral staining heterogeneity and the counting methods employed. Despite such problems, the Ki-67 index is an indispensable prognostic and therapeutic stratification marker for GEP-NENs, but Ki-67 cutoff values need to be refined with respect to the tumor type, location, and their response to therapy; this would require Ki-67 measurements for each individual tumor [8,10].

Currently, little is known about the potential diagnostic or clinical value of measuring systemic cytokine levels in patients with different types of neuroendocrine tumors or their value as diagnostic or prognostic indicators for pathohistological evaluations. Abnormal interactions between cytokines may be an overlooked mechanism for linking the development of different types of neuroendocrine tumors [11,12]. In this study, immunohistochemical expression of TNF-α, IL-6, IL-1β, and IL-2 in GEP-NENs was evaluated and correlated with the proliferation, tumor grade, tumor, node, metastasis (TNM), and outcomes.

## MATERIALS AND METHODS

GEP-NENs from 43 patients were retrieved from Ljudevit Jurak Pathology Department Tumor Registry, Zagreb, Croatia. Fourteen tumors originated from the stomach, 1 duodenal, 12 ileal, 12 appendiceal, and 4 colonic NENs were selected. One to seven blocks from each tumor were analyzed on hematoxylin and eosin (HE) slides. Tissue for light microscopy was fixed in 4% formaldehyde, embedded in paraffin using routine procedures, from which 5 µm thin sections were cut and stained with HE. All cases were reviewed by pathologists and diagnoses of GEP-NENs were verified with positive immunohistochemical reactions to synaptophysin and chromogranin. All cases meet the WHO criteria for the diagnosis of GEP-NEN. Tumors were divided into two groups according to a proliferation index using a Ki-67 threshold value set at 10%. Representative blocks (1 or 2) for each case were selected for immunohistochemical study.

The following primary monoclonal mouse antibodies were employed: TNF-α [MAB610] (R&D Systems, USA), IL-1β [11E5] (Santa Cruz Biotechnology, USA), IL-2 [N7.48A] (Santa Cruz Biotechnology, USA), and IL-6 [MAB2061] (R&D Systems, USA). DAKO EnVision™+System, HRP (DAB) was used to visualize positive reactions according to the manufacturer’s instructions. The slides were counterstained with hematoxylin. Appropriate negative and positive controls (small intestine mucosa for TNF-α and IL-1β, large intestine mucosa for IL-2, and appendix for IL-6) were employed. Immunohistochemical staining was evaluated for the whole tumor surface for each slide and tumor percentage was at least 50% of slide material. A slide was recorded negative (0) if there was no staining or staining was <5% tumor cells stained with medium to strong intensity; weak (1) for staining up to 25% tumor cells; moderate (2) 25–50% tumor cells; and strong (3) >50% tumor cells [12]. TNF-α was analyzed as a membranous reaction and IL-1β, IL-2, and IL-6 as cytoplasmic reaction. Two pathologists evaluated immunohistochemical findings and disagreements were resolved by joint reevaluation.

### Statistical analysis

The data were analyzed using IBM SPSS Statistics for Windows, Version 19.0. (IBM Corp., Armonk, NY, USA). Chi-square tests were used to analyze differences in the morphologic criteria and immunohistochemical staining patterns and their intensity. Fisher’s exact tests were used for dichotomous variables. Student’s *t*-test was used to compare the means of Ki-67 percentages. A *p* value <0.05 was considered statistically significant.

## RESULTS

The pathohistological samples of 43 patients (20 females and 23 males; mean age of females at diagnosis 47.35 years; and males 52.56 years) were analyzed in this study. Grading was carried out according to the WHO 2010 classifications [7]. Our samples contained 22 G1, 14 G2, and 7 G3 GEP-NENs. Females with G3 tumors were diagnosed at a mean age of 58.4 years and males at a mean age of 70 years. Females with G2 tumors were diagnosed at a mean age of 53.57 years and males at a mean age of 48.25 years, while females were diagnosed with G1 tumors at a mean age of 35 and males at a mean age of 51.08 years. Out of the 43 patients, most were diagnosed with NEN G1 (22; 51.2%). On average, patients had NENs of 12.7 years (SD = 3.59). At the time of our immunohistochemical evaluation, 27/43 (62.8%) patients were alive; for 9 patients, their survival status was unknown.

### Morphologic characteristics

**FIGURE 1:**
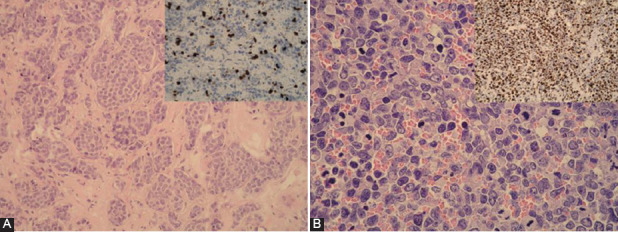
(A) Neuroendocrine neoplasia (NEN, G2) with islands, trabeculae, or sheets of monotonous cells with pink granular cytoplasms and round-oval stippled nuclei with minimal pleomorphism (hematoxylin and eosin [HE] ×200); inside psc: Ki-67×400~10%; group 1 for this study; (B) neuroendocrine neoplasia (NEN, G3) with large cells, eosinophilic cytoplasm, nuclear pleomorphisms, and hyperchromasia, prominent nucleoli, and numerous mitoses (HE ×400); inside psc: Ki-67×400 assessed 80%; group 2 for this study.

Most of the tumors in group 1 (Ki-67 ≤10%) had predominantly islands, trabeculae, or sheets of monotonous cells with a pink-purple granular cytoplasm and round-oval stippled nuclei, small nucleoli, minimal pleomorphisms, and no necrosis (Figure 1A). Tumors showing organoid arrangements with cells larger than those in small cell carcinomas, with nuclear pleomorphism and hyperchromasia, prominent nucleoli, numerous mitoses, and tumor necrosis, with a Ki-67 index of more than 10% were placed in a second group (Figure 1B).

### Immunohistochemistry findings

**FIGURE 2:**
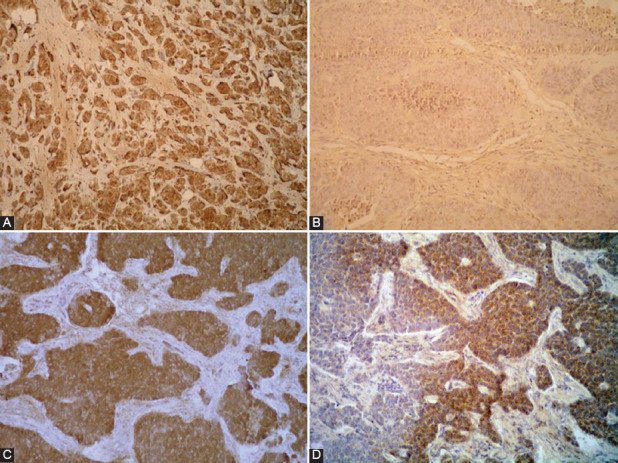
Immunohistochemical staining: (A) tumor necrosis factor alpha (TNF-α), more than 50% positive tumor cells (×200); (B) interleukin (IL)-1β, negative staining (×400); (C) IL-2, more than 50% positive tumor cells (×400); (D) IL-6, more than 50% positive tumor cells (×400).

Immunohistochemical expression of TNF-α was increased in tumor groups with high proliferation (*p* = 0.034, Table 1). About 75% of NENs with more than 25% positive cells in the >10% Ki-67 group had increased TNF-α levels, as opposed to 31.4% in the Ki-67 ≤10% tumor group. In the same group (Ki-67 ≤10%), 68.6% of tumors displayed low TNF-α expression (up to 25% of cells were positive). IL-6, IL-1β, and IL-2 displayed similar immunohistochemical expression in both groups, although expression between the ILs varied. Most GEP-NENs displayed high expression of IL-6 and reduced expression of IL-1β and IL-2. IL-6 expression increased with tumor grade; however, while close to setup p value, this measurement was not statistically relevant. Immunohistochemical staining is presented in Figure 2. Immunohistochemical expression of TNF-α positively correlated with death outcomes (*p* = 0.016, Table 2) and did not correlate with other examined markers. About 83.3% of patients with tumors displaying TNF-α expression in more than 25% of cells died as a course of the disease. Survivors had a fewer number of cells positive for TNF-α compared to patients with fatal outcomes (*p* = 0.023) and those lost to follow up (*p* < 0.001, Table 2).

**TABLE 1:**
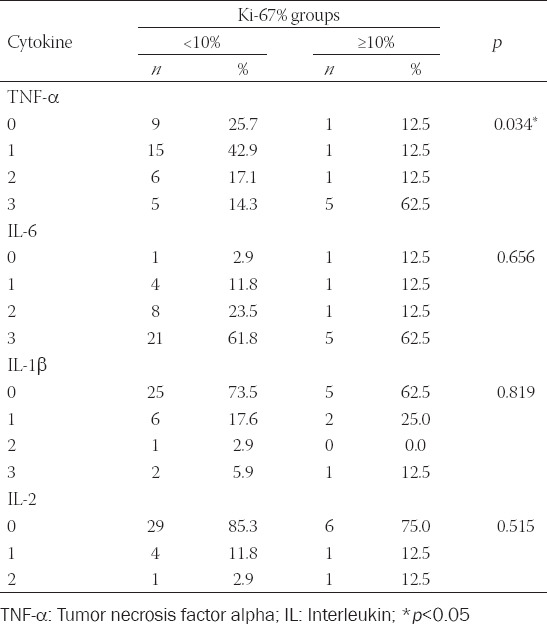
Immunohistochemical expression of proinflammatory cytokines according to Ki‑67 cutoff groups

**TABLE 2:**
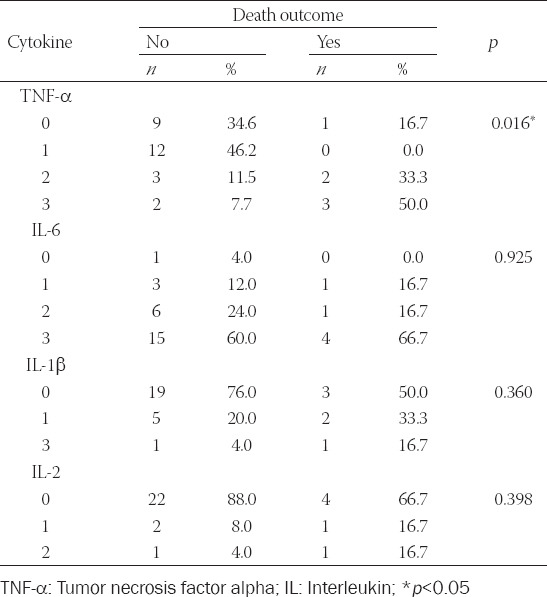
Immunohistochemical expression of proinflammatory cytokines and outcomes

## DISCUSSION

Nowadays, it is generally accepted that proinflammatory microenvironments are important drivers of all tumors; thus, substantial efforts have been devoted to the biochemical and immunological cytokine network interactions that promote the progression of tumors. Several multicenter studies have demonstrated that the presence of various polymorphisms in genes coding for ILs, such as IL-1, IL-6, and/or IL-8, is strongly associated with increased risks of cancer development [11,12]. There is extensive evidence that the neuroendocrine system (network of neuroendocrine cells) influences the function of the immune system; however, little is known about the potential diagnostic and clinical values derived from measuring systemic cytokine levels in patients with different types of neuroendocrine tumors, as well as their potential diagnostic abilities during the pathohistological evaluation of tumors [12-14].

Overall, cytokines are divided into proinflammatory groups such as TNF-α, IL-1β, IL-2, and IL-6, which play a role in initiating chronic inflammation and activating the NF-κB pathway and are strongly linked to cancer growth. Cytokine genes commonly have polymorphisms that are functionally neutral, but different single nucleotide polymorphisms (SNPs) have been identified that influence cytokine gene expression and function; consequently, tumor progression and host antitumor immunity. In our previous study, we demonstrated that changes in SNPs for TNF-α and IL-2 are associated with GEP-NET development. In addition, serum levels of these cytokines were more sensitive markers for both functioning and non-functioning GEP-NETs when compared to standard neuroendocrine markers such as CgA [15]. Despite all efforts, CgA is still the leading marker for routine diagnostics and monitoring of GEP-NENs, despite its relative lack of sensitivity or specificity [15-18].

GEP-NENs occur more frequently in chronic inflammation. Previous studies demonstrate that enteroendocrine cells can be hyperstimulated by chronic inflammation, which leads to their hyperplasia and neoplastic transformation [19]. Studies of neuroendocrine tumors imply important roles for cytokines such as IL-1 in directing cancer cells during neuroendocrine differentiation. Our previous studies revealed the involvement of IL-6 and IL-1β in the development of NENs. According to SNP analyses, IL-6 –174 CG and GG genotype carriers and IL-1β –511/+3954 CTCC carriers are at risk of developing non-functional NENs, while IL-1β –511/+3954 CTCT carriers are prone to development functional NENs [17,19]. Moreover, IL-6 GG genotype correlates with IL-6 serum levels that are significantly higher in patients with non-functioning NENs. Neuroendocrine neoplasms are densely vascularized and express hypoxia-inducible factor (HIF)-1α and vascular endothelial growth factor A (VEGF-A) [19]. In contrast to many types of tumors, where loss of differentiation accompanies increases in microvessel density, such GEP-NENs are paradoxically less vascularized, probably due to a defect in the HIF-1/VEGF-A pathway [19,20].

Therefore, unraveling the mechanisms of NENs pathogenesis may shed light on the molecular heterogeneity of neoplasms. Our results suggest that individual patterns of lesions may provide new tools for better diagnostics and targeted therapeutics. In our study, we analyzed the immunohistochemical expression of various cytokines that are known to create protumor proinflammatory microenvironments; IL-1β, IL-2, IL-6, and TNF-α. Our results demonstrate that neuroendocrine tumors have increased TNF-α protein levels which corresponds to higher tumor grades and proliferation rates (i.e., less differentiated tumors positively correlated with death outcomes). Most GEP-NENs had high expression of IL-6 and low expression of IL-1β and IL-2. Paradoxically IL-6 had reduced expression in high-grade tumors, although this result had a borderline *p* value.

Overall, our data indicate a role of inflammation in GEP-NEN carcinogenesis and the potential usefulness of cytokines as biomarkers; this may also help to refine our understanding of proinflammatory protumoral microenvironments in future studies.
